# Delivering on the Promise: Innovative Therapies and the Quest for (Real) Patient Benefit

**DOI:** 10.1097/HS9.0000000000000298

**Published:** 2019-09-12

**Authors:** Robin Doeswijk

**Affiliations:** European Hematology Association (EHA), The Hague, The Netherlands

**Figure:**
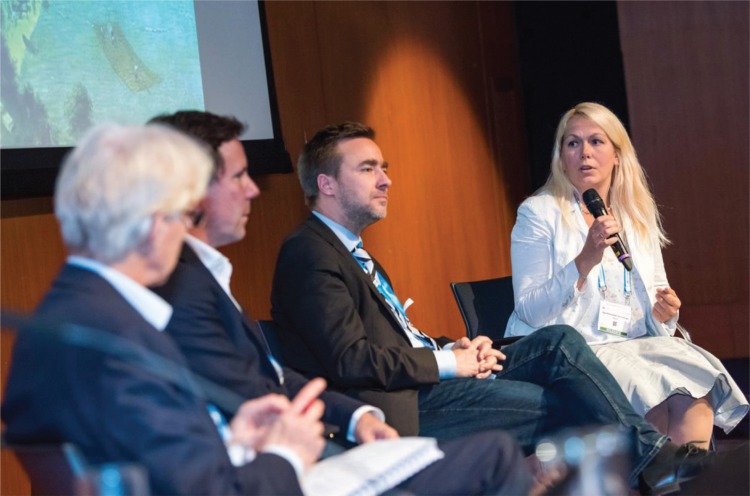
PHOTO: Olga Kholmanskikh of the Belgian medicines agency FAHMP discussing regulation of personalized medicine trials with fellow panel members (left to right) Prof. Ulrich Jäger (Medizinische Universität Wien/EHA European Affairs Committee), Johannes Pleiner-Duxneuner (Roche) and Jan Geissler (CML Advocates Network).

The EHA-Patient Joint Policy Symposium at EHA24 (Amsterdam, June 15) offered a day of stakeholder sessions on key policy and regulatory topics.^1^ In 5 sessions, mixed panels of hematologists, patient advocates, industry representatives and regulators treated an equally mixed audience to unfiltered views and lively debate.

## Innovative therapies: access, benefit and expectations

While novel cell and gene therapies are very promising, their introduction raises the question how patients can benefit, given the complexity, budgetary burden and uncertainty about the long-term effectiveness of these treatments. Speakers in the morning sessions set out looking for answers, and did some expection management along the way.

### How to ensure patient access to innovation and affordability?

Not surprisingly, the panel members addressing the question at the heart of this session did not come up with a unified answer. Francesco Pignatti (EMA) reminded the audience that neither innovation nor excellence are requirements for market authorization, but that this is based on safety and efficacy. Decisions on medical benefit and prescription are up to doctors and patients; the role of EMA is to make sure they can make these decisions based on the best available information.

The fact that a mandate has its inherent limitations was also evident in the two contributions on Health Technology Assessment (HTA). Patient advocate Zack Pemberton-Whiteley stressed that patient involvement in HTA must be combined with real influence on decision-making. EUnetHTA, represented by Anne Willemsen, considers input from patients essential, especially in the scoping phase. However, efforts to involve patients have so far had only limited success. Patient advocates in the audience mentioned several reasons for this, including lack of resources, COI issues and skepticism about the value of their contribution. Willemsen strongly defended the separation between joint clinical assessments, as piloted by EUnetHTA, and national-level appraisals, while Pemberton-Whiteley argued for reimbursement decisions to be made at the European level.

Market access specialist João Carapinha discussed concepts of ‘value-based’ versus ‘fair’ pricing. A believer in value-based pricing (VBP) – in his opinion, prices should not be fixed but always negotiable – he nonetheless warned policy makers against implementing VBP models, for lack of resources. ‘Who pays?’ remains the inevitable question.

### Patient-reported outcomes

Now that the importance of patient-reported outcomes (PRO) is broadly recognized, the four speakers in this session focused on their implementation. Patient advocate Giora Sharf discussed the use of PRO in clinical trials. The perception of the impact of PRO tends to differ between doctors and patients, but there is little doubt that they play a valuable role in stimulating discussion between the providers and the receivers of care. Tom Coats (King's College London) addressed the role of technology in hematology and cited “powerful studies” that suggest that PRO supports clinical decisions in cancer, with very good input even from computer-inexperienced patients.

Mark Skinner from the Institute for Policy Advancement in Washington, DC, is equally convinced that “patients can influence the clinical pathway”, with the help of methodologically sound PRO tools, such as the one he presented, PROBE (PRO, Burdens & Experiences). Another insightful contribution was provided by Sarah Liptrott of the Hematology Nurses & Health Care Professionals group (HNHCP). Nurses play a prominent role in the interaction between physicians and patients, and are convinced that PRO are beneficial also for physicians as it positively affects their satisfaction levels, efficiency and time. Interestingly, HNHCP has developed disease-specific PRO measures, such as for sickle cell disease.

### Managing the hype on CAR T-cell therapy

In front of an overflowing room – as befitted a session dedicated to a hype – Hermann Einsele (University of Würzburg) and Natacha Bolaños (Lymphoma Coalition) led a spirited discussion on CAR T-cell therapy.^2^ Patients, doctors, nurses and an EU policymaker shared their optimism and concerns, in varying dosages, for the implementation of this revolutionary therapy. To Brian Koffman, a doctor who became a CLL patient and who was an early recipient of CAR T-cell therapy, the balance is positive: while the side effects are tough, the benefit for the patients that respond is real and significant.

Ananda Plate of Myeloma Patients Europe agreed that CAR T-cell therapy is very promising, although in myeloma “it does not look like a miracle yet”. She called on all stakeholders to agree on a combined set of tools – communication, PRO/Quality of Life (QoL), health economics, protocol – that help achieve meaningful benefit for patients. Not knowing exactly what to expect from CAR T treatments causes uncertainty among both patients and nurses. As Mairead Ní Chonghaile of HNHCP asserted, “there is an awful lot of doubt and fear among healthcare professionals – all of them”.

Jan van de Loo, cancer expert of the European Commission, views health inequalities across Europe and pricing as the main issues. The Commission acknowledges that Europe lags behind China and the US, but is faced with limited funding from Member States. It focuses its efforts on comprehensive uptake of CAR T-cell therapy in health systems, EU added value, partnerships, and fostering innovation. The last round of Horizon 2020 calls will offer “lots of opportunities for cell therapy”, on top of the two specific CAR T-cell topics in the most recent IMI2 call.

## Regulating innovative therapies

Later in the day, speakers at the EHA-Patient Joint Policy Symposium highlighted the need for adaptation of regulatory processes to facilitate true patient-centered, personalized medicine.

### Personalized medicine trials

Leading the call for regulatory changes and flexibility was Ulrich Jäger from the Medical University of Vienna, member of EHA's European Affairs Committee. New regulations are needed to enable the tailored decision-making and personalization of trials that are necessitated by the combination of high-risk patients, treatment failures, rare diseases and novel target or drug discoveries. Adaptation is also required from pharmaceutical companies, in order to make multi-drug trials possible. An encouraging example is Roche experimenting with 10-drug trials. Challenges for companies include financial and legal risks, unwanted stimulus for off-label use, and, as Johannes Pleiner-Duxneuner of Roche Austria put it, “the need to turn real-world data into real-world evidence” which requires the combination of databases.

Jan Geissler (CML Advocates Network) stressed the need to involve patients in all the difficult decisions on ethics, regulation and data protection. “If it is all for the patient, why are we not asked what an acceptable risk is?” He also pointed out that while new regulation may be necessary, much can be done with existing mechanisms such as the Clinical Trials Regulation, Adaptive Pathways and PRIME. Clinical assessor Olga Kholmanskikh Van Criekingen of Belgian medicines agency FAHMP agreed with fellow panel members that new trial designs could help increase the use of biomarkers and personalize treatments, that big data is crucial, and that patient input “along the continuum” is important. She signaled a number of risks, however, including increased operational complexity and challenges to safety oversight, data integrity and transparency. Widely diverging patient opinions on data sharing and usage are a complicating factor.

There was agreement among the members of this panel addressing ‘personalized medicine trials’ that the data ownership question is crucial. Registries are seen as part of the solution, and hopes are that valuable lessons can be drawn from the HARMONY project (a public-private partnership, funded by the Innovative Medicines Initiative, which aims to leverage Big Data to advance the study and treatment of hematologic malignancies).^3^

### Raising the bar for drug approvals

The most debated issue on this day was formulated, somewhat provocatively, by session chair Ton Hagenbeek (Amsterdam UMC/EHA European Affairs Committee): “EMA is bound by its mandate, but as a physician I say: there should be a real medical benefit before a medicine can be approved.” Raising the bar for drug approvals, by adding a ‘real added value for patients’ criterion to efficacy and safety, was not something that could, or should, be expected of EMA, as Francesco Pignatti explained. Not regulators, but doctors and patients should decide on medical benefit. “A paternalistic approach of raising the bar for all is not the answer.” And: “There is not a single bar we can all agree on”.

Patient advocate Piarella Peralta (Inspire2Live) proposed refocusing the discussion to “raising the bar for patient care with quality of life”. This requires re-centering healthcare around the “magic combination of patients, clinicians and researchers”. Responding to Prof. Hagenbeek challenge that industry trials result in many ‘me-too’ drugs that are good for industry profits but of little added value to patients, Takeda's Kelly Page acknowledged industry can do better. She insisted, however, that “our responsibility as industry is to run the best studies we can”. Page expressed confidence that “patient involvement throughout the development continuum can help ensure value-added outcome”.

There was consensus among panel and audience members on the need for meaningful involvement of patients in decisions on benefit, and for alignment of all stakeholders to determine patient-relevant endpoints. In a somewhat prickly exchange on the merits of ‘me-too’ drugs, however, patients in the room reminded everyone that patients are not a monolithic group with identical preferences. What is of negligible benefit to some, may be invaluable to others.

## Footnotes

1 For a full overview of the program, see https://ehaweb.org/assets/Uploads/EHA-Patient-Joint-Policy-Symposium-full-overview-FINAL.pdf.

2 *EHA Guidance Document: The process of CAR-T cell therapy in Europe*, HemaSphere: August 2019 - Volume 3 - Issue 4 - p e280.

3 See https://www.harmony-alliance.eu. EHA is a partner of the HARMONY Alliance.

